# Dataset of working conditions and thermo-economic performances for hybrid organic Rankine plants fed by solar and low-grade energy sources

**DOI:** 10.1016/j.dib.2016.03.002

**Published:** 2016-03-09

**Authors:** Domenico Scardigno, Emanuele Fanelli, Annarita Viggiano, Giacobbe Braccio, Vinicio Magi

**Affiliations:** a School of Engineering, University of Basilicata, Potenza 85100, Italy; b Technical Unit for Trisaia Technologies (UTTri), ENEA, Rotondella 75026, Italy

**Keywords:** Organic Rankine cycle, Low-grade energy, Solar energy, Thermo-economic analysis

## Abstract

This article provides the dataset of operating conditions of a hybrid organic Rankine plant generated by the optimization procedure employed in the research article “A genetic optimization of a hybrid organic Rankine plant for solar and low-grade energy sources” (Scardigno et al., 2015) [1]. The methodology used to obtain the data is described. The operating conditions are subdivided into two separate groups: feasible and unfeasible solutions. In both groups, the values of the design variables are given. Besides, the subset of feasible solutions is described in details, by providing the thermodynamic and economic performances, the temperatures at some characteristic sections of the thermodynamic cycle, the net power, the absorbed powers and the area of the heat exchange surfaces.

**Specifications Table**

**Table:** 

Subject area	*Engineering*
More specific subject area	*Thermodynamics*
Type of data	*Tables*
How data was acquired	*Numerical simulations based on FluidProp 9.0 database*
Data format	*Raw*
Experimental factors	*No pretreatment of data was performed*
Experimental features	*The simulations have been performed on MS Excel spreadsheets by means of VBA macros*
Data source location	*Florence, Italy*
Data accessibility	*Data is with this article*

**Value of the data**

• This data provides several working conditions of a hybrid power plant by considering 24 working fluids and several design parameters.
• The reader can choice among the different solutions by evaluating their thermodynamic and economic performances.
• The reader can compare these solutions with other layouts fed by the same sources and by employing the same working fluids.
• These solutions can be compared with other solutions that employ working fluids not considered in this study.
• The readers can exclude any particular solution under consideration by checking it on the unfeasible solution dataset.

## 1 Data

This article provides the feasible and unfeasible operating conditions of a hybrid organic Rankine plant, given in Ref. [1]. The conditions have been selected by using the NSGAII (Non-dominated Sorting Genetic Algorithm) [2]. The data provides, for each feasible solution, four design parameters, thermo-economic performances and other dependent variables, such as temperatures, powers and sizes of the heat exchangers. The solutions characterized by the best thermo-economic performances are given in Ref. [1], so they are not included in the dataset.

The unfeasible solutions are characterized by a set of working conditions that give an unphysical behavior of the plant. These solutions are reported in a distinct dataset.

## 2 Experimental design, materials and methods

In the following, the configuration of the power plant is firstly described, then the design variables are given, the solution procedure and the model used to assess the plant cost are described, and finally the three performance indicators are discussed.

### 2.1 Power plant configuration

**Fig. 1:**
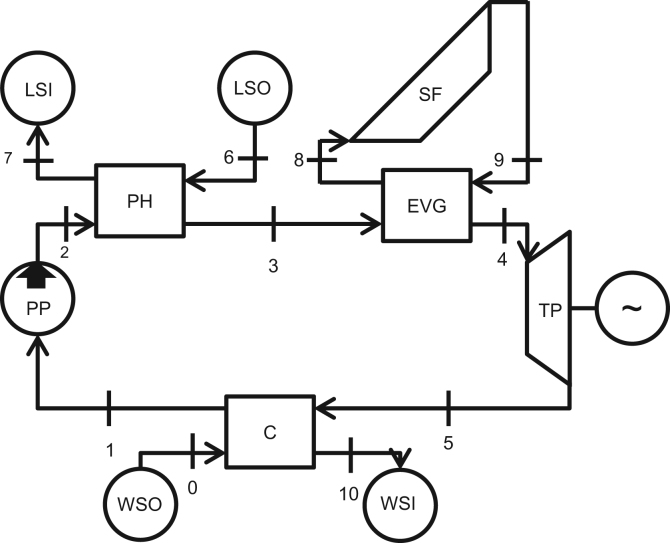
Power plant configuration [1].

The layout of the power plant is shown in Fig. 1. The working fluid is compressed by the pump PP and absorbs heat at first by the low-grade energy source in the heat exchanger PH and afterward by the solar field SF in the heat exchanger EVG. Then the working fluid is expanded in the turbine TP in order to obtain mechanical power. Finally, the fluid is condensed by the condenser C and returns to the pump.

The preheater is fed by a mass flow rate of water, equal to 1 kg/s, at a temperature of 90 °C. The pressure drops in the heat exchangers are neglected. The solar field is made up of evacuated tube collectors, where the heat transfer fluid, assumed to be water in this study, can work at a maximum pressure of 6 bar and a maximum temperature of 150 °C. The ambient air has a temperature of 20 °C and a pressure of 1 bar.

### 2.2 Design and dependent variables

The design variables are: i) the working fluid, which is selected among the fluids listed in Table 1, ii) the evaporating pressure pev, iii) the condensing pressure pcond, iiii) the maximum temperature of the collector thermal fluid T9, iiiii) the variable ∆*Tdes* that rules the following relations:

(1) T4=T9−∆Tdes

(2) ∆Tpinch,EVG=∆Tde

(3) ∆Tpinch,PH=∆Tdes

(4) $$T3=\{\begin{array}{l} \min(T6-∆\mathrm{Tdes},T\left(\mathrm{pev}\right))\;\mathrm{if}\mathrm{subcritical}\\ T6-∆\mathrm{Tdes}\;\;\mathrm{if}\mathrm{supercritical} \end{array}$$

(5) ∆Tpinch,C=∆Tdes

where ∆*Tpinch* is the pinch temperature. For a subcritical working condition, Eq. (4) prevents the working fluid from evaporating, being *T*(pev) the phase change temperature of the working fluid at the pressure pev.

The dataset provides, for the feasible solutions, the following dependent variables: the temperature (*T*3) of the working fluid at the outlet section of the preheater, the temperatures (*T*7 and *T*10) at which water is discharged into the environment by the preheater and by the condenser, the minimum temperature (*T*8) of the collector thermal fluid, the net power (Wnet), the input powers of the heat exchangers (QPH and QEVG), the size of the heat exchangers and of the solar collectors.

### 2.3 Solution procedure

After setting the values of pcond, pev, *T*9, ∆*T*des and the specific working fluid, the thermodynamic cycle is solved by using the solution procedure given in the following items:

• *T*1 lies on the saturated liquid curve at the pcond pressure;
• *T*2 is a function of *T*1, pcond, pev and of the isentropic efficiency of the pump, set to 0.75;
• *T*3 is is calculated from Eq. (4);
• *T*7 is a function of *T*2, *T*3, *T*6 and ∆*Tpinch*, *PH*=∆*Tdes*;
• The mass flow rate $\dot{m}$ of the working fluid is calculated from the law of conservation of energy applied to the heat exchanger PH;
• *T*4 is calculated by applying the definition of ∆*Tdes* given in Eq. (1);
• *T*8 is a function of *T*9, *T*4, *T*3 and ∆*Tpinch*, *EVG*=∆*Tdes*;
• The mass flow rate $\dot{m}\mathrm{coll}$ of the solar collector fluid is calculated from the law of conservation of energy applied to the heat exchanger EVG;
• *T*5 is computed from *T*4, pcond, pev and from the isentropic efficiency of the turbine, set to 0.8;
• *T*10 follows from *T*0, *T*1, *T*5 and ∆*Tpinch*, *C*=∆*Tdes*;
• Finally, the mass flow rate of the water through the condenser is calculated from the conservation of energy.

The thermodynamic properties of the fluids are assumed according the Refprop 9.0 database [3].

### 2.4 Cost evaluation

The most expensive components of a standard ORC plant are the heat exchangers [4]. The heat exchanger area is estimated by calculating the heat transfer coefficient by means of two different models depending on whether the working condition is supercritical or subcritical. The heat transfer coefficient for an exchanger in supercritical working condition is calculated by means of the Petukhov-Kranoschekov׳s correlation [5]. The heat transfer coefficient in subcritical working condition is computed by means of the Dittus-Boelte׳s correlation for the single phase region and the Wang-Touber׳s correlation for the two phase region [6]. In the dataset the heat exchange area of the condenser C, of the preheater PH and of the exchanger EVG are named AC, APH and AEVG, respectively. Then, the cost of each heat exchanger in the year of 2014 is obtained by applying a correlation that takes into account the heat exchange area and the working pressure [7]. The routines for the evaluation of the heat exchange surface area are written on spreadsheets in Visual Basic For Applications.

Another expensive component is the solar field. The solar collector area, Acoll, is given by:

(6) $$A\mathrm{coll}=\frac{\dot{Q}\mathrm{EVG}}{\eta \mathrm{coll}*\mathrm{Gsun}}$$

where $\dot{Q}\mathrm{EVG}$ is the thermal power transferred to the working fluid by the heat exchanger EVG, Gsun is the solar irradiance, set to 930 W/m^2^, and ηcoll is the collector efficiency, computed as [8]:

(7) $$\eta \mathrm{coll}=0.718-0.974\frac{\mathrm{Tm}-T0}{\mathrm{Gsun}}-0.005\frac{{\left(\mathrm{Tm}-T0\right)}^{2}}{\mathrm{Gsun}}$$

where *Tm* is the average temperature of the collector thermal fluid and *T*0 is the ambient temperature. The cost of the solar field is estimated by assuming a price of 202.35 $/m^2^.

### 2.5 Thermodynamic and economic performances

The performances of the solutions are assessed by means of two thermodynamic indicators and an economic indicator. The first one is the first law efficiency:

(8) $$\eta I=\frac{\dot{W}\mathrm{net}}{\dot{m}\mathrm{water}\left(h6-h7\right)+\mathrm{Gsun}*\mathrm{Acoll}}$$

where $\dot{W}\mathrm{net}$ is the net power output, $\dot{m}\mathrm{water}$ is the mass flow rate of water in the preheater and h6 and h7 are the water enthalpy at the inlet and outlet of the preheater, respectively.

The second indicator is the second law efficiency:

(9) $$\eta \mathrm{II}=\frac{\dot{\mathrm{Wnet}}}{\dot{E}\mathrm{water}+\dot{E}\mathrm{sun}}$$

(10) $$\dot{E}\mathrm{water}=\dot{m}\mathrm{water}\left[h6-h7-T0\left(s6-s7\right)\right]$$

(11) $$\dot{E}\mathrm{sun}=\dot{Q}\mathrm{sun}(1-\frac{4T0}{3\mathrm{Tsun}})$$

where $\dot{E}\mathrm{water}$ is the waste water exergy, *s* is the entropy, $\dot{E}\mathrm{sun}$ is the solar exergy [9], $\dot{Q}\mathrm{sun}$ is solar radiation incident on the collector, *Tsun* is the equivalent black-body sun temperature, set to 5800 K.

The economic indicator is the LEC (Levelized Energy Cost) defined as:

(12) $$\mathrm{LEC}=\frac{I+\mathrm{OM}+F}{\mathrm{Egen}}$$

(13) $$I=\left(\mathrm{Ccoll}+\sum \mathrm{Cexc}\right)*\frac{i{\left(i+1\right)}^{y}}{{\left(i+1\right)}^{y}-1}$$

where *I* is the annualized present value of total investment cost, *OM* is the annualized present value of the operating and maintenance cost, *F* is the present value of the annual fixed cost, Egen is the annual electricity output, *Ccoll* is the cost of the solar field, ∑Cexc is the total cost of the three heat exchangers, i is the interest rate, set to 5%, and *y* is the plant lifetime, set to 20 years. The operating and maintenance cost is set to 5% of the total cost of the heat exchangers and to 3% of the cost of the solar field. The fixed cost is neglected. The annual electricity output is computed for 3000 working hours/year.

## Figures and Tables

**Fig. S1:**
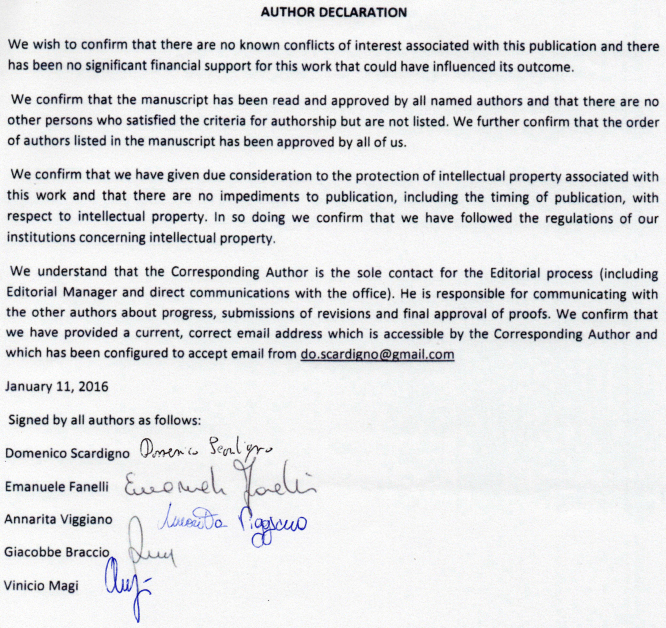


**Table 1:** List of working fluids.

• R32 • R41 • R125 • R134a • R143a • R152a • R218 • R227ea	• R236ea • R236fa • R245fa • R245ca • Cyclopropane • Propane • RC318 • Butane	• Isobutane • CF3I • C5F12 • Pentane • Isopentane • Neopentane • Hexane • Propylene
